# A Handheld IoT Vis/NIR Spectroscopic System to Assess the Soluble Solids Content of Wine Grapes

**DOI:** 10.3390/s25144523

**Published:** 2025-07-21

**Authors:** Xu Zhang, Ziquan Qin, Ruijie Zhao, Zhuojun Xie, Xuebing Bai

**Affiliations:** College of Enology, Northwest A&F University, Xianyang 712100, China

**Keywords:** wine grape, visible-near-infrared spectroscopy, IoT, soluble solids content, data optimization

## Abstract

The quality of wine largely depends on the quality of wine grapes, which is determined by their chemical composition. Therefore, measuring parameters related to grape ripeness, such as soluble solids content (SSC), is crucial for harvesting high-quality grapes. Visible–Near-Infrared (Vis/NIR) spectroscopy enables effective, non-destructive detection of SSC in grapes. However, commercial Vis/NIR spectrometers are often expensive, bulky, and power-consuming, making them unsuitable for on-site applications. This article integrated the AS7265X sensor to develop a low-cost handheld IoT multispectral detection device, which can collect 18 variables in the wavelength range of 410–940 nm. The data can be sent in real time to the cloud configuration, where it can be backed up and visualized. After simultaneously removing outliers detected by both Monte Carlo (MC) and principal component analysis (PCA) methods from the raw spectra, the SSC prediction model was established, resulting in an $R_{V}^{2}$ of 0.697. Eight preprocessing methods were compared, among which moving average smoothing (MAS) and Savitzky–Golay smoothing (SGS) improved the $R_{V}^{2}$ to 0.756 and 0.766, respectively. Subsequently, feature wavelengths were selected using UVE and SPA, reducing the number of variables from 18 to 5 and 6, respectively, further increasing the $R_{V}^{2}$ to 0.809 and 0.795. The results indicate that spectral data optimization methods are effective and essential for improving the performance of SSC prediction models. The IoT Vis/NIR Spectroscopic System proposed in this study offers a miniaturized, low-cost, and practical solution for SSC detection in wine grapes.

## 1. Introduction

It is now widely accepted that wine quality primarily depends on the chemical characteristics of the wine grapes (*Vitis vinifera* L.) [1]. The quality of grapes is determined by their chemical composition, making information about their ripening status a key factor in producing high-quality wines [2]. The chemical components of grapes include the soluble solids content (SSC), which refers to the total amount of dissolved solids present in a given volume of fruit juice, encompassing components such as carbohydrates, organic acids, proteins, lipids, and minerals [3]. One degree Brix of SSC is approximately equivalent to 10 g/L of sugar, and is approximately equivalent to 1.8% of potential alcohol content [4]. Therefore, SSC not only serves as a key indicator of grape maturity and sugar concentration at harvest but is also commonly utilized to predict the potential alcohol content in the resulting wine. SSC can be assessed using refractometers, chromatographs, biosensors, and hydrometers; however, these methods often fail to meet the simultaneous requirements of low cost and rapid detection [5]. In addition, these are destructive methods because they generally require the sample to be crushed to extract the juice for measurement, particularly in the case of solid samples [6]. This limitation makes them less suitable for large-scale, rapid, or real-time analysis.

There is a growing interest in noninvasive measuring methods, such as Visible–Near-Infrared Spectroscopy (Vis/NIR) [7,8]. Vis/NIR technology is optical-based and noninvasive, enabling the non-destructive and real-time analysis of the grape chemistry. Vis/NIR combines spectral measurements in the visible (Vis) light (400–700 nm) range and the NIR (700–2500 nm) range [9]. The maturity and quality of grapes are reflected not only in surface color but also in internal components such as SSC. When incident light strikes the grape surface, part of the reflected light forms diffuse reflection, consisting of both surface-reflected light and light that is backscattered from shallow internal tissues [10]. The visible spectrum primarily captures color-related attributes of the sample, while the near-infrared spectrum provides information about its internal chemical composition [11]. Another feature of Vis/NIR is its rapid detection capability; the time for performing a measurement can be no more than one minute, which is much faster than methods such as refractometers, chromatographs, biosensors, and hydrometers [5]. Many studies have been conducted using benchtop commercial spectrometers to measure the SSC of fruits. Aparatana et al. [12] used a DS2500 spectrometer (FOSS, Hillerød, Denmark) to measure the SSC of sugarcane. Nevertheless, the bulky size and substantial weight of benchtop spectrometers restrict their applicability outside of laboratory environments. Commercial portable spectrometers with smaller size, such as the USB2000+ (Ocean Optics, Dunedin, FL, USA), have been employed to determine the SSC in grapes [13] and apples [14]. Additionally, a FieldSpec 4 ASD spectrometer (Analytical Spectral Devices, Boulder, CO, USA) was used to determine the SSC in several cultivars of wine grapes [15].

With the development of microelectronics technology and sensor technology, the research and application of miniaturized Vis/NIR devices are being valued. A series of thumb-sized spectrum sensors have been developed to detect the quality of fruits. The C11708MA sensor (Hamamatsu Photonics, Shizuoka, Japan) has been integrated into handheld spectrometers to evaluate the SSC of fruits such as kiwis [16], peaches [17], and yellow nectarines [18], while the C14384MA-01 sensor (Hamamatsu Photonics, Shizuoka, Japan), which has the same spectral response range but a more compact design, has been applied to measure the SSC of mangoes [19], mandarins [20], and apples [21]. Miniaturized spectrometers developed using smaller-sized, lower-power multispectral sensors have also been reported. The AS7262 and AS7263 sensors (AMS-Osram, Premstätten, Austria) were combined to extend the spectral response range and were applied to assess the SSC of bananas [22,23] and grapes [24,25].

Despite offering significant advantages in terms of cost and weight, miniaturized Vis/NIR devices still show performance gaps compared to benchtop commercial spectrometers, particularly in aspects such as sensitivity, resolution, and signal-to-noise ratio. Optimizing spectral data is a more cost-effective strategy without improving hardware components. There are many unstable factors in the process of spectral detection, and the spectral data can be affected by interference from information unrelated to the properties of the target sample. To improve the signal-to-noise ratio, spectral data preprocessing is necessary [10,26,27]. Wang et al. [28] compared the effectiveness of various preprocessing methods for handling rice Vis/NIR spectral data, including multiplicative scatter correction (MSC), standard normal variate (SNV), normalization, moving average smoothing (MAS), Savitzky–Golay smoothing (SGS), first derivative (FD), and second derivative (SD). Due to factors such as instrument drift, unstable light sources, surface contamination of samples, and improper operations, the measured spectral or chemical values may deviate significantly from their true values. These abnormal samples can generally be categorized into spectral value outliers and chemical value outliers. The identification and removal of such outliers are crucial steps in improving the accuracy and reliability of visible/near-infrared (Vis/NIR) detection [26,27]. The Monte Carlo (MC) outlier detection approach was applied to identify potential outliers in the samples measured using Vis/NIR spectroscopy [29,30]. Wang et al. [23] and Zhang et al. [31]. employed principal component analysis (PCA) to detect the outliers in Vis/NIR spectral data collected from bananas. Among all spectral variables, redundancy, multicollinearity, and spectral overlap are inevitable. This spectral overlap complicates the isolation of unique spectral features related to target analytes, increasing model complexity. Characteristic wavelength algorithms can efficiently reduce the dimensionality of spectral data and eliminate redundant information. This not only simplifies model complexity and reduces training time but also mitigates the risk of overfitting [10,26,27]. Feature selection was conducted using the successive projections algorithm (SPA) and uninformative variable elimination (UVE) to identify the key wavelengths of Vis/NIR spectroscopy data for grapes, although the SPA selected fewer characteristic wavelengths, and the model built with the wavelengths selected by UVE demonstrated better predictive performance [32].

Therefore, the goal of this research is to develop a low-cost, handheld spectral device that is affordable for growers and other users, and to pave the way for monitoring further chemical parameters of grape ripening from veraison to harvest. To achieve this objective, this study develops a low-cost spectral sensing system capable of directly acquiring Vis/NIR reflectance measurements from grapes in the field. It is designed as an IoT device functioning as a terminal node, with the potential for extension into smart vineyard and winery systems. Within this context, the specific objectives of this study are to describe the design and development of the IoT-based spectral sensing system, validate the usability of the proposed spectral sensing system through data acquisition experiments, and identify and analyze key characteristics of the optical signals collected under both laboratory and field conditions, developing a representative prediction model for SSC based on laboratory data.

## 2. Materials and Methods

This research developed a detection device for the SSC of grapes based on Vis/NIR spectral technology. The development of the grape SSC detection device mainly included hardware system design and software design. The hardware system design included a development board module, sensor module, power module, data transmission module, storage module, input module, and display module. The software system development involved both the embedded programming of the Arduino board and the customized development of an open-source IoT platform. Functions such as multi-channel spectral acquisition, result display, local storage, and cloud data backup and visualization were realized.

### 2.1. Hardware Design

To reduce cost and simplify maintenance, commercially available components were selected whenever possible. To facilitate broader adoption and ease of use, both size and price were also considered during the design process. The following sections provide a detailed description of the selected components:

- Arduino MKR ZERO (Arduino LLC, Monza, Italy): The Arduino MKR ZERO board was chosen for its low cost, compact size, and reliable performance. It uses the Arm Cortex-M0 32-bit SAMD21 processor and also integrates a micro SD card holder with dedicated SPI (Serial Peripheral Interface). In addition, the board provides an extra SPI and one IIC (Inter-Integrated Circuit) interface for connecting external modules. The board runs at 3.3 V and it has an onboard voltage regulator to convert from 5 V to 3.3 V which satisfies the power supply requirements of both 5 V and 3.3 V components simultaneously.
- AS7265X Triad Spectroscopy Sensor (AMS-Osram, Premstätten, Austria): The AS7265X chipset consists of three sensor devices: AS72651 with master capability, AS72652, and AS72653. The multispectral sensors can be used for spectral identification in a range from visible to NIR. Each of the three sensor devices has 6 independent on-device optical filters whose spectral response is defined in a range from 410 nm to 940 nm with an FWHM of 20 nm. The Triad is combined with a 405 nm UV (Ultraviolet), 5700 k white, and 875 nm IR LEDs to illuminate and test various surfaces for light spectroscopy.
- DR150 (USR, Jinan, China): The DR150 is a high-speed, low-delay, and user-friendly 4G Cat 1 DTU (Data Transfer Unit) with a built-in IoT SIM card. In transparent transmission mode, it enables serial devices to transmit data to a designated server and receive data from the server, which is then forwarded to the serial interface. In addition to hardware support, the manufacturer also provides an IoT cloud platform, allowing users to rapidly develop customized IoT applications.
- SD card (SanDisk, Milpitas, CA, USA): Thanks to the built-in micro SD (Secure Digital) card slot on the Arduino MKR ZERO, storage module can be easily prepared by simply inserting an SD card into the board. Once inserted, data can be read from or written to the card. After data recording is complete, the SD card can be removed and accessed on a computer for further analysis or storage.
- Li-Po battery (CKE, Jiangmen, China): A single-cell 5 V 18,650 lithium polymer (Li-Po) battery is used to supply DC (direct current) power to other modules, with a maximum supported power output of 10 W and a capacity of 3400 mAh. This configuration ensures long-term operation of the device, with an estimated cycle life of approximately 800 charge–discharge cycles.
- IPS LCD screen (ZJY, Beijing, China): A 1.54-inch color liquid crystal display (LCD) screen with an integrated font chip was selected. It features a resolution of 240 × 240 RGB, offering clear and vivid display performance.
- Push-button switch (Youxin, Shenzhen, China): A non-latching push-button switch is used, with the signal taken from the OUT port. When the button is pressed, a high-level signal is output; when released, the output remains at a low level.
- TPE enclosure (Sangong, Shenzhen, China): A TPE (thermoplastic elastomer) enclosure measuring approximately 223 × 192 × 65 mm was used to house and protect the internal modules. Its pistol-shaped design enhances grip comfort and operational convenience.

Table 1 lists the models and prices of modules integrated into the Vis/NIR multispectral devices: the total hardware cost is slightly less than 1000 CNY, which is significantly lower than the cost of commercial handheld Vis/NIR spectrometers and approximately one-third of the cost of the digital refractometer used in this study. With a weight of only around 500 g, the device is compact and ergonomically designed for convenient one-handed operation.

**Figure 1:**
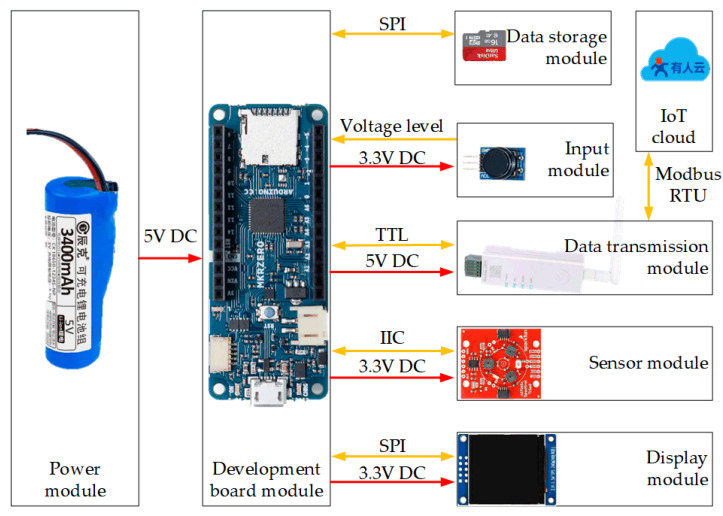
Connection diagram of the Vis/NIR multispectral device components.

Each module is connected to the corresponding pins on the development board using DuPont cables to establish power supply and data communication. The DR150 module operates at 5 V, while the other components operate at 3.3 V, which is converted from the 5 V supply through an onboard voltage regulator. A common ground is shared among all components to ensure stable operation and signal integrity. The AS7265X sensor communicates with the development board via the IIC interface, receiving measurement commands and returning spectral data accordingly. The development board sends display commands to the LCD screen with the open SPI interface, and the results are presented in textual and numerical formats. Upon receiving the storage command, the spectral data are written to the SD card inserted in the slot via a dedicated SPI interface. The data is transmitted to the DR150 DTU through the TTL (Transistor Transistor Logic) interface, which then sends it to the IoT cloud platform using the Modbus RTU (Remote Terminal Unit) protocol. Figure 1 illustrates the connections among the components of the multispectral device, the orange arrows represent signal transmission, while the red arrows indicate power supply.

**Figure 2:**
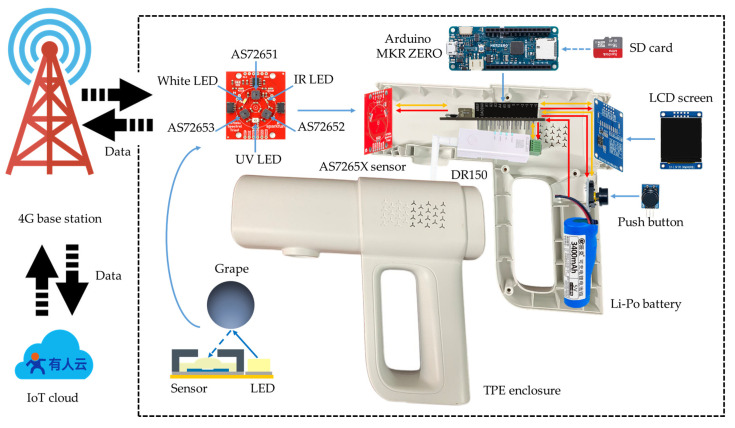
Scheme of the multispectral device hardware structure and IoT system architecture.

The multispectral sensor device was composed of different elements that were assembled inside a TPE enclosure. The objective was to develop a tool that is reliable and easy to operate under field conditions by non-specialized personnel. The DTU sends data to the nearby 4G base station, which is ultimately transmitted to the IoT cloud platform server. On the cloud platform, users can manage the wireless gateway and visualize the data. Figure 2 illustrates the scheme of the multispectral device hardware structure and IoT system architecture.

### 2.2. Software Design

#### 2.2.1. Embedded Programming Design

**Figure 3:**
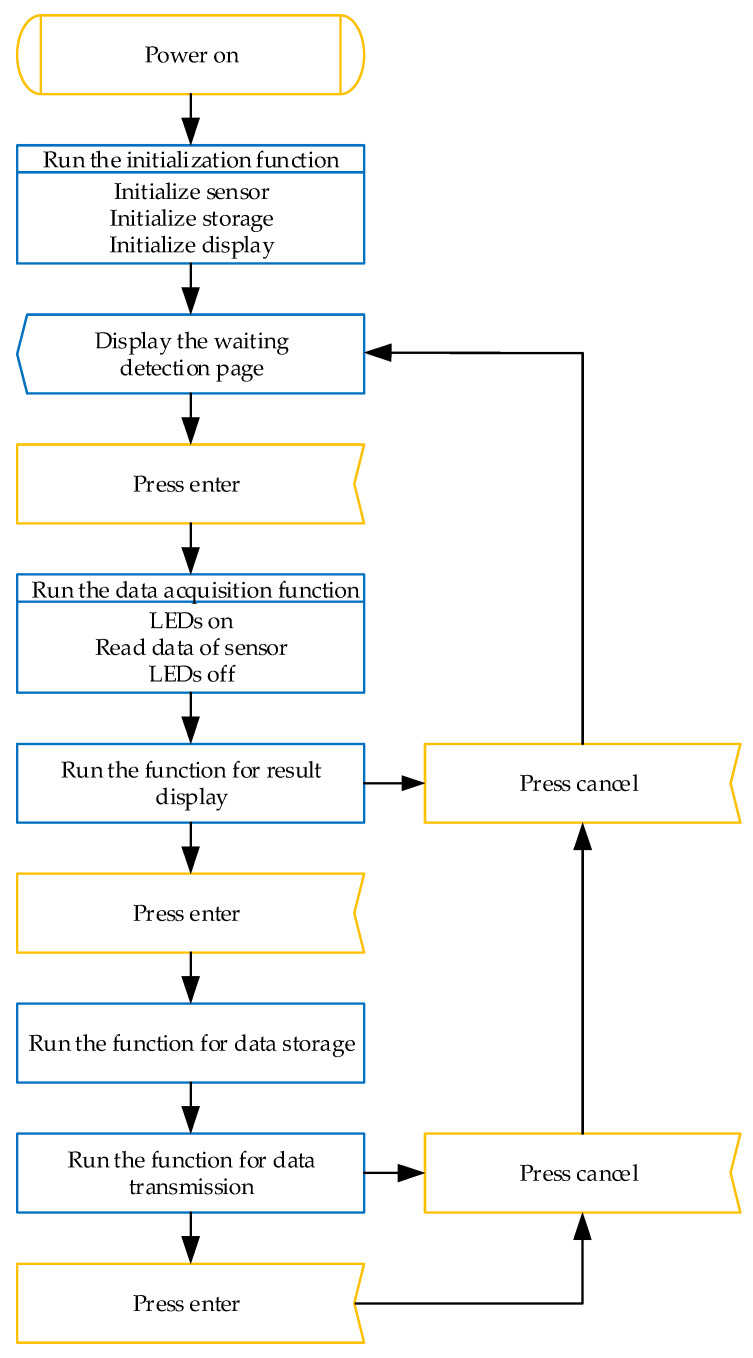
Flowchart of the multispectral device operation.

The embedded program of the Arduino board was developed on Arduino IDE. A USB-A to Micro-USB data cable was used to connect the computer and the development board, enabling serial monitoring during sketch debugging and uploading the compiled program to the board. The code primarily encompasses the initialization configuration and functional implementation of the sensor, display, storage, and transmission modules. Figure 3 shows the running logic of the Arduino sketch while the multispectral device was operated; the orange rectangles represent user operations, while the blue rectangles indicate the execution of programs on the development board. The device operation can be described in the following steps:

1. Insert an SD card with a capacity not exceeding 16 GB into the card slot. Align the grape sample with the spectroscopy sensor assembled at the front port of the device, then press the self-locking switch button on the battery power cable to start the device.
2. After the device is powered on, the development board automatically executes the initialization function, during which communication parameters for the display, sensor, and storage modules are configured, connection statuses are verified, test tasks are performed, and finally the initialization result are returned. Meanwhile, the wireless transmission module automatically completes parameter initialization, searches for available networks, and connects to the 4G Cat-1 network.
3. After the device initialization is completed, the LCD screen displays a ‘Waiting for Detection’ interface. When the user presses the confirmation button, the development board initiates the sensor data acquisition process. The LED lamp is first activated to illuminate the surface of the grape sample and diffuse reflection occurs. A portion of the reflected light that carries information about the grape’s quality enters the spectroscopy sensor and is measured. Upon completion of data acquisition, the LED is turned off.
4. After acquiring data from the sensor, the development board executes the result display function, sequentially presenting the reflectance values of the 18 channels. When this set of data needs to be saved and transmitted, the confirmation button is pressed. The device saves the spectral data in a .txt file and transmits the data to the IoT cloud platform in transparent mode.
5. If the device is no longer in use while powered on, press the same switch to turn it off. Users can view data tables and download data to a local computer through the IoT cloud platform. Alternatively, they can remove the SD card and copy the stored .txt file to a computer using a card reader.

#### 2.2.2. IoT Cloud Configuration Design

For the purpose of real-time backup and cloud-based sharing of spectral data, the data was transmitted through the wireless communication module to the USR IoT cloud, which is an open-source platform provided by the module manufacturer and integrates essential functionalities such as configuration services and centralized data management. Based on the data transmission protocol and the structure of the data blocks, virtual devices were then added to establish a virtual spectrometer and ensure the correct mapping of each spectral variable. Finally, using the graphical tools provided in the configuration management interface, the cloud configuration was developed to support device configuration management, real-time data visualization, and the display of information including text, images, and geolocation.

### 2.3. Data Aquisition

The sampling area was located at the Rongzi chateau in the Loess Plateau region (Xiangning, Shanxi, China; 110°49′37.700″ E, 36°1′20.921″ N) during the harvest period in September 2023, which was delayed due to the plateau climate. Sampling was performed on multiple batches of Cabernet Sauvignon grapes (*Vitis vinifera* L.) that were immediately delivered to the preprocessing facility after harvest. 

To ensure the stability, repeatability, and accuracy of the spectral data acquired by the AS7265X sensor, and to minimize the influence of ambient light and sensor drift, before the acquisition of spectral data, dark calibration was conducted under a fully light-shielded environment, while white calibration was conducted using a standard white reference panel. All measurements were performed under controlled lighting conditions to eliminate external light interference. For each sample, three spectral acquisitions were performed, and the average reflectance of the three spectra was used as the representative value. A total of 18 spectral variables—410, 435, 460, 485, 510, 535, 560, 585, 610, 645, 680, 705, 730, 760, 810, 860, 900, and 940 nm—were collected by the Vis/NIR multispectral device. The spectral values were used as feature variables for the SSC prediction model. A total of 120 samples were collected and detected within one day.

The widely used accurate but destructive refractometric method is used to detect SSC in grapes. A digital refractometer with temperature correction (PAL-Grape Must, ATAGO, Tokyo, Japan) was employed to measure the SSC of the grapes. The refractometer was set to zero by adding distilled water to the sample stage before use. Before each measurement, the residual juice was removed by rinsing with distilled water and thoroughly wiping the sample stage clean. Each measurement was performed in triplicate and the results were expressed in °Brix. Grape juice was manually squeezed and dropped on the refractometer. The SSC values served as the target variable for the SSC prediction model.

### 2.4. Data Optimization and Modeling Methods

To ensure the accuracy of prediction model, the removal of outliers is a critical step in data analysis. In this study, the MC outlier detection method was employed to identify anomalous data points in both the feature (X) and response (Y) datasets. This method relies on repeated random sampling and statistical analysis, evaluating each sample’s deviation from the population based on the distribution of means and standard deviations across a large number of simulations [30]. For comparison, PCA was also applied to the raw and preprocessed spectral data to investigate the underlying data structure and variability. Outliers and sample trends were identified through the evaluation of Hotelling’s T^2^ statistics at a 5% significance level [23]. The modeling results based on the original dataset, the datasets with outliers removed using MC and PCA individually, and the dataset with outliers removed by both methods were compared. The dataset that achieved the best performance was then selected for further spectral preprocessing.

To improve the accuracy of spectral analysis, it is essential to mitigate the effects of stray light, noise, and baseline drift, which frequently interfere with raw spectral signals. Therefore, appropriate preprocessing of the original spectra is necessary before modeling. In this study, eight spectral preprocessing methods were evaluated to determine the most effective approach. These methods were categorized into four groups: baseline correction, including the FD and SD; scattering correction, including MSC and SNV; smoothing, including SGS and MAS; and scaling, including normalization and autoscaling [33]. The modeling performance of spectra preprocessed using eight different methods were compared, and the method that yields the best results was selected for subsequent characteristic wavelength selection.

To improve the generalization capability and stability of the predictive model, effective wavelength selection techniques were applied to eliminate irrelevant variables and reduce spectral redundancy. By selecting wavelengths that were highly correlated with the target characteristics, these methods helped to minimize the risk of overfitting and mitigate spectral overlap. Specifically, UVE and SPA were used to extract the most informative wavelengths from the full spectral range, thereby reducing data dimensionality, enhancing computational efficiency, and improving model accuracy [34,35]. The modeling performance based on wavelengths selected by UVE and SPA will be compared to determine the more effective feature selection method.

Partial Least Squares (PLS) regression is a statistical method used to investigate the relationship between predictor variables and response variables. It is especially effective for datasets characterized by multicollinearity and limited sample sizes. To ensure representative sampling for model development, the SPXY (Sample set Partitioning based on joint X–Y distances) method was utilized to partition the dataset. Specifically, 75% of the samples were allocated to the calibration set, and the remaining 25% were allocated to the validation set [36]. PLS was employed to correlate the optimized multispectral dataset with the SSC dataset. The performance of the PLS model was evaluated by calculating the coefficient of determination for both the calibration set ($R_{C}^{2}$) and the validation set ($R_{V}^{2}$), along with the root mean square error for calibration (RMSEC) and validation (RMSEV). All spectral preprocessing, outlier detection, characteristic selection, and model development procedures were programmed in MATLAB/Simulink R2020b (MathWorks, Natick, MA, USA).

## 3. Results

### 3.1. IoT Cloud Configuration

**Figure 4:**
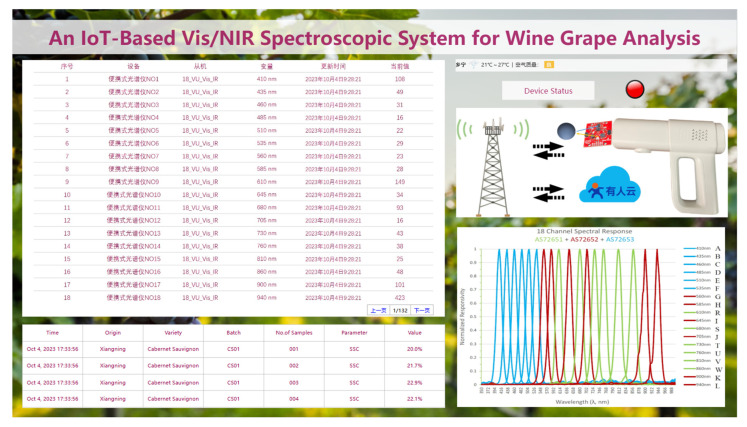
IoT cloud configuration for Vis/NIR Spectroscopic System.

The collected spectral data were transmitted in real time to the USR cloud platform by a 4G network, enabling cloud-based configuration management and data visualization through a custom-developed interface. Figure 4 illustrates the interface of the cloud configuration system. On the left side of the interface, a table displayed the real-time spectral reflectance values of each wavelength, which automatically refreshed upon receiving new data. The lower-left section showed the SSC values of measured grapes, which were manually entered. The right side of the interface presented geographical location and weather information, along with a schematic diagram of the system architecture and spectral variable curve plots.

### 3.2. Raw Spectroscopy

**Figure 5:**
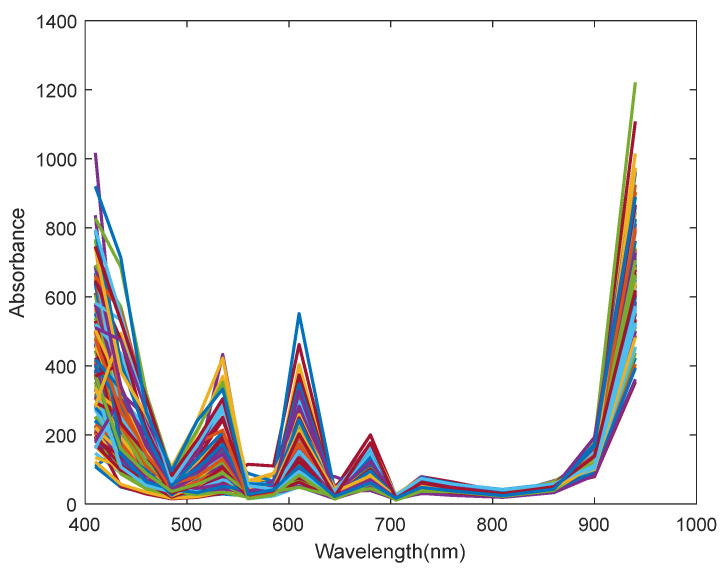
Raw absorbance spectra obtained by Vis/NIR multispectral device.

The SSC values and spectral data of 120 grape samples were measured under controlled low-light conditions. The collected spectral data are presented in Figure 5. Distinct reflectance peaks were observed between 410–680 nm and 860–940 nm, covering the visible spectrum and extending into the red-edge [37]. In the visible range, two absorbance peaks were identified: one around 500 nm, associated with anthocyanin content, and another near 680 nm, corresponding to chlorophyll content. A spectral trough appeared at approximately 705 nm, followed by a gradual increase in the near-infrared region, which can be attributed to the fourth and third overtones of C–H bonds and the third overtone of O–H bonds [14].

### 3.3. Outlier Detection

**Figure 6:**
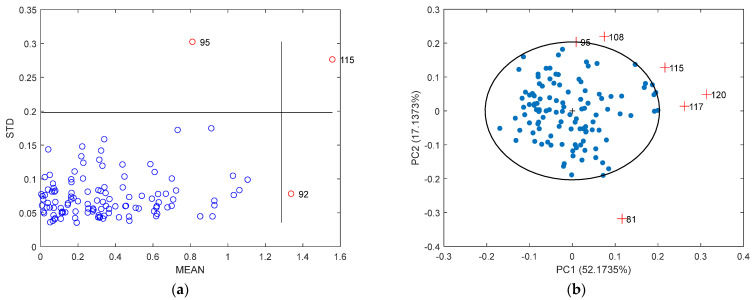
Outlier detection map of the original spectral dataset: (**a**) outlier detection results using the MC method; (**b**) outlier detection results using the PCA method.

In this study, the MC and PCA outlier detection method was applied to the collected spectral data and SSC measurements. As illustrated in Figure 6a, the MC approach identified samples with unusually high means and standard deviations as outliers, which are marked in purple, while the normal samples are shown in blue. Consequently, the samples labeled 92, 95, and 115 were removed based on the results of the outlier detection. These samples were located in the fourth, second, and first quadrants, respectively, as defined by the horizontal standard deviation and vertical mean threshold lines. As shown in Figure 6b, the outliers are indicated by red plus, while the normal samples are shown by blue dots. The distribution of samples within the 95% confidence ellipse following PCA processing reveals that samples 81, 95, 108, 115, 117, and 120 fall outside the ellipse and are thus identified as outliers. Notably, except for sample 81, the remaining five outliers are located in the first quadrant. Among all the outliers, samples 95 and 115 were detected as outliers by both methods.

**Figure 7:**
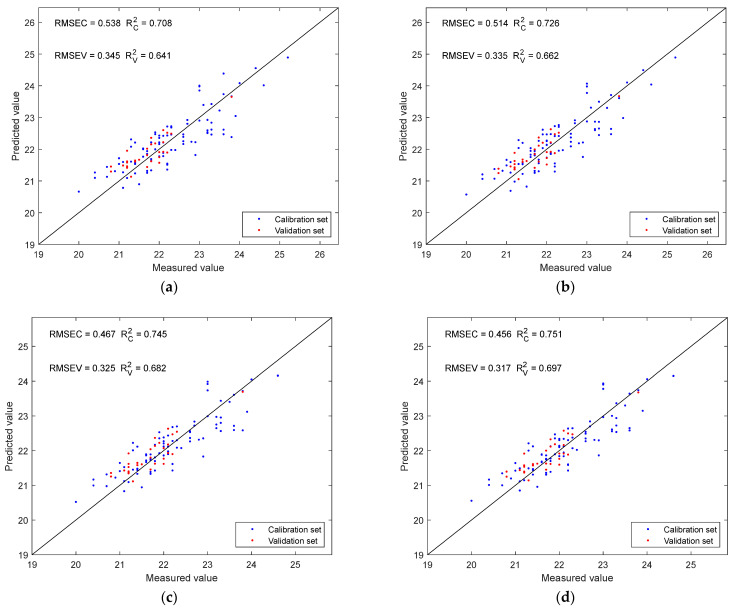
The fitting plots of the prediction models established using the four different datasets: (**a**) prediction results of the original dataset; (**b**) prediction results of the datasets with outliers removed by the MC method; (**c**) prediction results of the datasets with outliers removed by the PCA method; (**d**) prediction results of the dataset with outliers removed by both methods.

The SPXY method was employed to divide the dataset into a calibration set and a validation set at a ratio of 3:1. SSC prediction models were developed based on four datasets: the original dataset, datasets with outliers removed using the MC or PCA method individually, and a dataset with outliers removed by both methods. Model performance was compared to evaluate the effectiveness of each outlier detection approach, as shown in Figure 7. The model based on the original dataset yielded an $R_{V}^{2}$ of 0.641. After removing outliers using the MC and PCA methods separately, $R_{V}^{2}$ values improved to 0.662 and 0.682, respectively. The highest $R_{V}^{2}$ of 0.697 was achieved when outliers identified by both methods were excluded. Table 2 presents the typical statistical results of the calibration and validation sets after the removal of all identified outliers. 

### 3.4. Spectral Data Preprocessing

**Figure 8:**
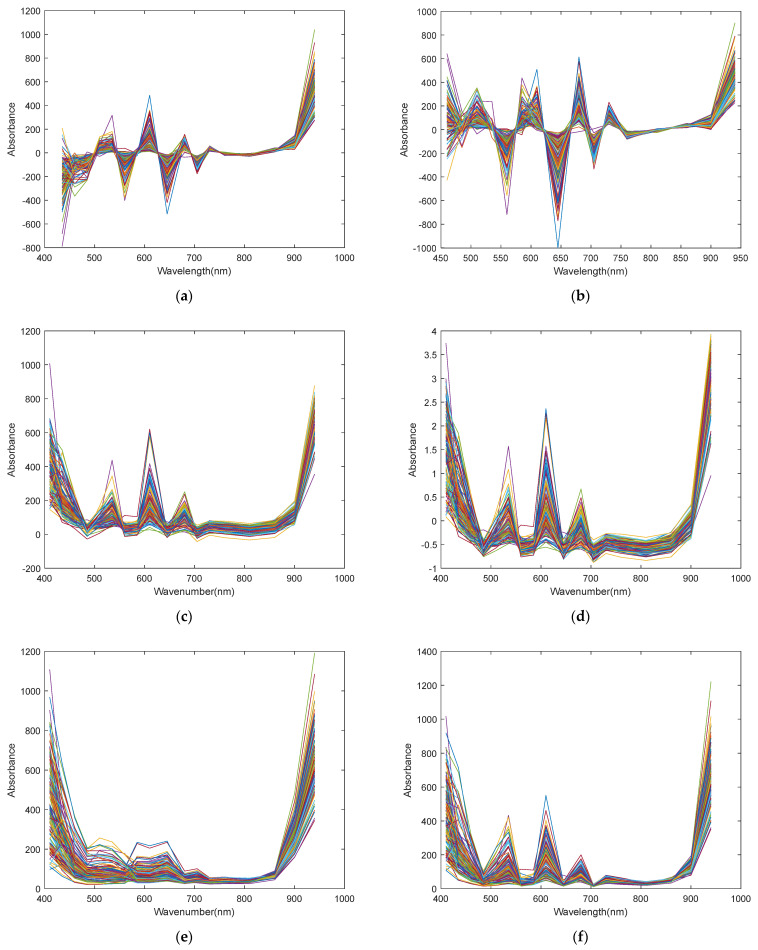

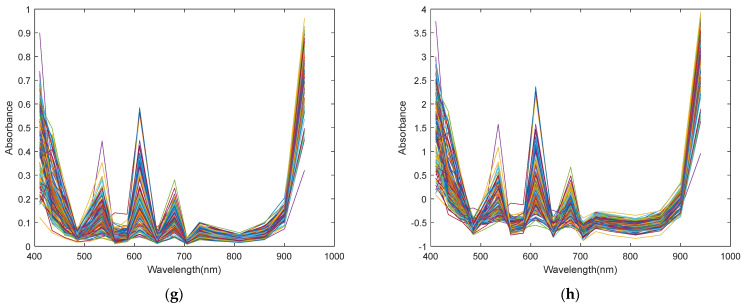
Figure of preprocessed spectra with different methods: (**a**) FD method; (**b**) SD method; (**c**) MSC method; (**d**) SNV method; (**e**) MAS method; (**f**) SGS method; (**g**) normalization method; (**h**) autoscaling method.

Figure 8 shows the spectral curves of the original data after being processed by each of the eight preprocessing methods. FD and SD were applied to eliminate baseline drift on the raw spectra. In Figure 8a, the positive peaks reflect the ascending trends of the original spectral curves, whereas the negative valleys correspond to the descending trends. The waveform in Figure 8b exhibits a similar overall pattern to that in Figure 8a. MSC and SNV were applied to correct scattering effects on the original spectra. As shown in Figure 8c,d, the overall waveform is generally consistent with that of the original spectra; however, the spectral curves exhibit greater vertical dispersion, which is noticeable in the 700–900 nm wavelength range. MAS and SGS were applied to reduce noise. In Figure 8e, MAS causes the spectral peaks to become lower and broader, whereas in Figure 8f, SGS preserves the original waveform more effectively. Normalization and autoscaling were applied to standardize the data scale. In Figure 8g, the response values were scaled to the range of 0 to 1 through normalization, while in Figure 8h, autoscaling adjusted the response values to range from −1 to 4.

**Figure A1:**
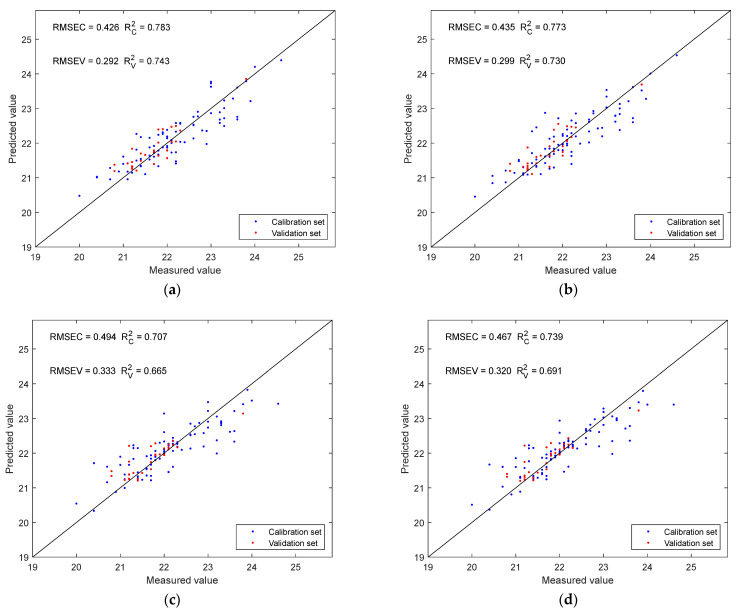

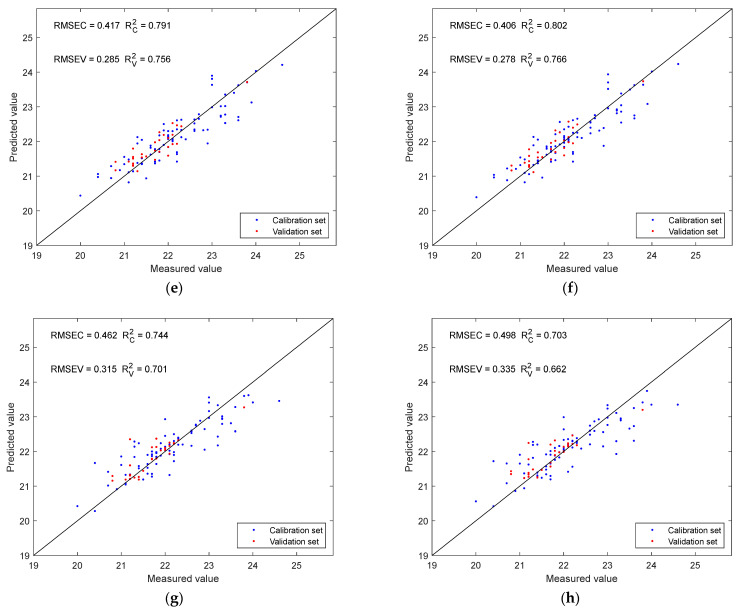
The fitting plots of the prediction models established with preprocessed spectra: (**a**) FD method; (**b**) SD method; (**c**) MSC method; (**d**) SNV method; (**e**) MAS method; (**f**) SGS method; (**g**) normalization method; (**h**) autoscaling method.

Table 3 compares the performance of models built using spectral data preprocessed with each of the eight methods. The model built using data preprocessed with MSC and autoscaling achieved an $R_{V}^{2}$ value of approximately 0.66, which is comparable to that obtained using the raw spectra. When spectral data were preprocessed by SNV and normalization, the $R_{V}^{2}$ increased to around 0.70. Preprocessing with FD and SD further improved the model performance, resulting in an $R_{V}^{2}$ of approximately 0.74, which is higher than that of the model constructed from the dataset after removing all outliers. Notably, the application of the two smoothing methods led to a further increase in $R_{V}^{2}$, reaching approximately 0.76. Figure A1 presents PLS prediction plots based on eight preprocessing methods.

### 3.5. Characteristic Wavelength Selection

**Figure 9:**
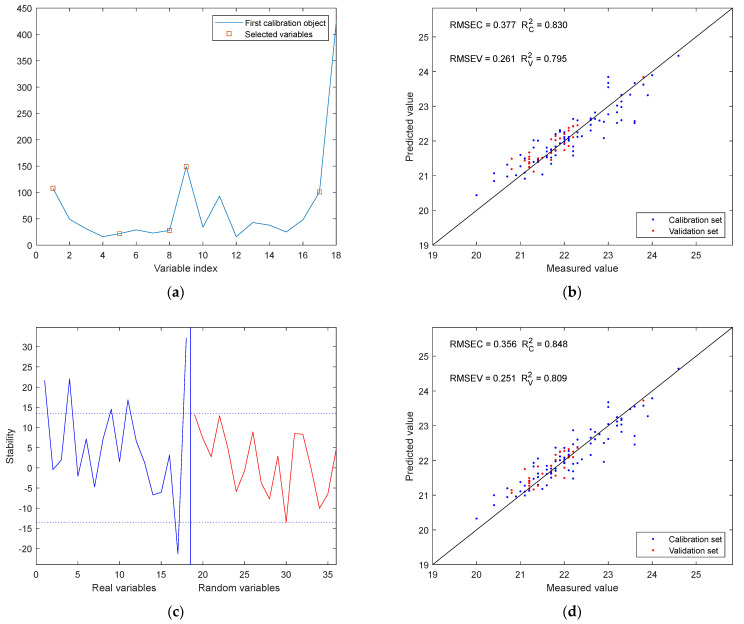
Characteristic wavelength selection and modeling results: (**a**) characteristic wavelength selected by SPA; (**b**) prediction model fitting plots based on characteristic wavelengths selected by SPA; (**c**) characteristic wavelength selected by UVE; (**d**) prediction model fitting plots based on characteristic wavelengths selected by UVE.

Each spectral variable carries a different amount of information. By applying the SPA and UVE, the characteristic wavelengths that contribute most significantly to the prediction of SSC in grapes were identified. Figure 9a shows that the spectral variables selected by the SPA method are 1, 5, 8, 9, and 17, corresponding to wavelengths of 410 nm, 510 nm, 585 nm, 610 nm, and 900 nm. As shown in Figure 9c, the blue line represents the stability of the real variables, while the red line indicates the stability of the random variables. According to the stability criterion, the UVE method selected variables 1, 4, 9, 11, 17, and 18, corresponding to wavelengths of 410 nm, 485 nm, 610 nm, 705 nm, 900 nm, and 940 nm. Reference [32] investigated the Vis-NIR spectra (400–1100 nm) of table grapes and identified key wavelengths using the competitive adaptive reweighted sampling (CARS) algorithm. The selected central wavelengths in their study included 400 nm, 500 nm, 600 nm, 700 nm, 800 nm, and 950 nm, which cover or are close to the characteristic wavelengths (410 nm, 610 nm, and 900 nm) identified in this work. This overlap indicates the rationality and consistency of the wavelength selection results. The wavelengths 410 nm, 610 nm, and 900 nm were selected by both methods, and the number of wavelengths selected by the two approaches differed by only one. Figure 9b, d present the prediction model fitting plots based on the characteristic wavelengths selected by SPA and UVE, respectively. The $R_{V}^{2}$ for the SPA-based model was 0.795, which is slightly lower than that of the UVE-based model at 0.809.

## 4. Discussion

In this study, a multispectral sensing device was developed by integrating the AS7265X sensor with other miniaturized, low-power, and cost-effective components. The device is capable of measuring 18 spectral variables across the Vis/NIR wavelength range, and is designed for assessing the SSC of wine grapes. The collected spectral data can be shared in real time to a cloud platform, facilitating remote monitoring and management. The commonly used VIS/NIR spectrometer USB2000+ (Ocean Optics, Dunedin, FL, USA) covers a spectral range of 350–1000 nm with a resolution of 1.5 nm. In comparison, the device developed in this study operates within a narrower range of 410–940 nm and has a lower resolution of 20 nm. This means that less spectral information is captured [38], which may explain the relatively low $R_{V}^{2}$ of only 0.641 for models based on the raw spectral data. Similarly, a relatively low coefficient of determination (R^2^) of 0.70 for SSC prediction was also reported in a related study [37]. In contrast, an $R_{V}^{2}$ of 0.87 was obtained using SGS-preprocessed spectral data for Cabernet Sauvignon with the USB2000+ spectrometer [13], whereas the present study achieved a slightly lower $R_{V}^{2}$ of 0.766.

Regarding the outlier detection results presented in Section 3.3, in the distribution plot of outliers detected by the MC method, the lower-left region represents normal samples. Sample 92, located in the lower-right quadrant, has a mean value exceeding the threshold and is therefore classified as a physicochemical outlier. Sample 95, found in the upper-left quadrant, exhibits a large standard deviation, indicating it is a spectral outlier. Sample 115, situated in the upper-right quadrant, shows abnormalities in both spectral and SSC values [39]. In the outlier distribution plot identified using the PCA method, the outliers 95, 108, 115, 117, and 120, located in the first quadrant (PC1 > 0, PC2 > 0) of the PLS score plot, represent samples with elevated spectral responses along both of the first two latent variables most strongly associated with SSC. This distribution may reflect either high sugar content or abnormal spectral intensities, potentially resulting from factors such as excessive surface gloss, uneven sample tissues, or instrumental variations [40].

Analyzing the preprocess results described in Section 3.4, an overall comparison shows that scatter correction and scale scaling did not significantly improve model performance, whereas baseline correction and smoothing methods obviously enhanced model performance. This indicates the raw spectra likely contained signal drift and random noise, with these two effective methods, respectively, improving spectral stability and signal-to-noise ratio [41]. Among the eight preprocessing methods, the model built using spectra preprocessed by SGS achieved the highest $R_{V}^{2}$ of 0.766.

Concerning the outlier detection results presented in Section 3.5, UVE retained more feature variables than SPA and yielded better model performance which is consistent with the prediction results reported in the reference [42]. This is because UVE focuses on identifying variables that contribute stable and significant information, rather than minimizing the number of variables or ensuring absolute independence. In contrast, SPA emphasizes minimizing collinearity among variables and tends to select a smaller number of mutually independent variables.

Among all modeling strategies evaluated in this study, the PLS model developed by integrating MC and PCA for outlier removal, SGS for spectral preprocessing, and UVE for feature selection exhibited the best predictive performance. This integrated approach achieved the highest $R_{V}^{2}$ = 0.809 and the lowest RMSEV = 0.251, confirming its effectiveness as the optimal modeling strategy.

## 5. Conclusions

In this study, a handheld spectral data acquisition device was successfully developed, along with a cloud-based configuration system built on an IoT platform. Spectral data acquisition was carried out by integrating and utilizing modules such as the AS7265X spectral sensor and the MKR ZERO board, followed by comprehensive optimization of the spectral data. The device offers several advantages, including portability, low cost, low power consumption, and network connectivity. However, its prediction performance based on raw data remains suboptimal due to the limited number of channels and low spectral resolution. Future research should consider improving the light source to enhance the device’s data acquisition efficiency and accuracy, and to support additional sampling modes such as transmission. In terms of data processing and modeling, more effective optimization algorithms and predictive models tailored to the characteristics of multispectral data should be investigated to improve prediction performance. These enhancements will help increase the device’s usability and reliability, providing more accurate and dependable theoretical and technical support for related studies.

## Figures and Tables

**Table 1:** Table of module models and prices.

Module	Model	Price (¥)
Development board	Arduino MKR ZERO	298
Sensor module	AS7265X Triad Spectroscopy Sensor	450
Data transmission module	DR150 DTU	117
Storage module	SD card	33.8
Power module	Li-Po battery	20
Display module	IPS LCD screen	27
Input module	Push-button switch	2.4
Connector	2.54 mm DuPont cable	10
Enclosure	TPE enclosure	15

**Table 2:** Statistical results of the SSC calibration and validation sets.

Set	No. of Samples	Min. (°Brix)	Max. (°Brix)	Mean (°Brix)	STD. (°Brix)
Whole set	113	20.00	24.60	22.03	0.86
Calibration set	83	20.00	24.60	22.15	0.91
Prediction set	30	20.80	23.80	21.69	0.58

**Table 3:** Comparison of PLS prediction models with eight different preprocess methods.

Preprocess Type	Method	$R_{C}^{2}$	RMSEC	$R_{V}^{2}$	RMSEV
Baseline correction	FD	0.783	0.426	0.743	0.292
	SD	0.773	0.435	0.730	0.299
Scattering correction	MSC	0.707	0.494	0.665	0.333
	SNV	0.739	0.467	0.691	0.320
Smoothing	MAS	0.791	0.417	0.756	0.285
	SGS	0.802	0.406	0.766	0.278
Scaling	Normalization	0.744	0.462	0.701	0.315
	Autoscaling	0.703	0.498	0.662	0.335

## Data Availability

The original data presented in the study are available upon request.

## Abbreviations

The following abbreviations are used in this manuscript:

SSC	Soluble Solids Content
SPI	Serial Peripheral Interface
IIC	Inter-Integrated Circuit
DTU	Data Transfer Unit
SD	Secure Digital
Li-Po	Lithium Polymer
LCD	Liquid-Crystal Display
TPE	Thermoplastic Elastomer
IoT	Internet of Things
MC	Monte Carlo
PCA	Principal Component Analysis
FD	First Derivative
SD	Second Derivative
MSC	Multiple Scattering Correction
SNV	Standard Normal Variate
SGS	Savitzky–Golay Smoothing
MAS	Moving Average Smoothing
UVE	Uninformative Variable Elimination
SPA	Successive Projections Algorithm
PLS	Partial Least Squares
SPXY	Sample set Partitioning based on joint X–Y distances
$R_{C}^{2}$	Coefficient of Determination for the Calibration Set
$R_{V}^{2}$	Coefficient of Determination for the Validation Set
RMSEC	Root Mean Square Error for Calibration Set
RMSEV	Root Mean Square Error for Validation Set
CARS	Competitive Adaptive Reweighted Sampling
DC	Direct Current
RTU	Remote Terminal Unit
UV	Ultraviolet

## Appendix A

Figure A1 shows the fitting plots of the prediction models established with spectra preprocessed by eight methods.
